# Internet Use and Self-Rated Health Among Older People: A National Survey

**DOI:** 10.2196/jmir.1311

**Published:** 2009-12-02

**Authors:** Enrique Gracia, Juan Herrero

**Affiliations:** ^2^University of OviedoOviedoSpain; ^1^University of ValenciaValenciaSpain

**Keywords:** Aged, computers, health, Internet, social class, socioeconomic status

## Abstract

**Background:**

Older people are among the segments of the population for which the digital divide is most persistent and are considered to be at risk of losing out on the potential benefits that the information society can provide to their quality of life. Little attention has been paid, however, to relationships between Internet use and actual indicators of health among older people.

**Objective:**

The aim of this study was to examine the association between Internet use and self-rated health among older people and determine whether this association holds independently of socioeconomic position.

**Methods:**

Data were from a survey about the digital divide and quality of life among older people in Spain that was conducted in 2008. The final sample consisted of 709 individuals and was representative of the Spanish adult population in terms of Internet use and sex across two age groups (55-64 and 65-74 years). Multivariate logistic regression analyses were performed to assess the relationship between Internet use and self-rated health.

**Results:**

Results initially showed a significant relationship between Internet use and poor self-rated health (Model 1, OR = 0.32, 95% CI 0.16-0.67, *P* = .002), suggesting that Internet users have better self-rated health than nonusers. This effect remained significant when other sociodemographic variables were entered into the equation (Model 2, OR = 0.39, 95% CI 0.18-0.83, *P* = .01; Model 3, OR = 0.41, 95% CI 0.19-0.87, *P* = .02). However, the significant relationship between Internet use and self-rated health disappeared once social class was considered (Model 4, OR = 0.61, 95% CI 0.27-1.37, *P* = .23).

**Conclusions:**

This study suggests that the use of the Internet is not a significant determinant of health among older people once the socioeconomic position of individuals is taken into account.

## Introduction

Older people are among the segments of the population with lower levels of Internet use—levels that decline sharply with advancing age [1-4]. For example, recent data from Europe indicate that 27% of people over age 54 and only 10% of people over 65 used the Internet, compared to 68% of those 16-24 [5].

The exclusion of older people from the information society is an issue of growing concern. For instance, the European Commission is developing a highly proactive agenda to break the barriers that prevent the older generation from fully embracing the information society and to promote the digital inclusion of older people [6,7]. Behind these efforts lies the idea that access to the information society can have a significant impact on the well-being and quality of life of older people. Access and use of the information society would contribute toward active aging and advancing health into old age by, for example, helping older people overcome isolation and loneliness, helping them keep in contact with family and friends by extending social networks, and facilitating the access and use of relevant information and services [3,7,8]. Little scholarly attention has been paid, however, to differences in health among older people who are users or nonusers of the Internet. This is an important issue to be examined given the efforts and investment that are being directed to promote e-inclusion among older people. For example, the European Commission i2010 Initiative on e-Inclusion acknowledges the persistent digital divide among older people, and it proposes to target this group of the population since they are considered at risk of losing out on potential benefits to their quality of life [6]. Efforts such as this should, however, be based on careful research rather than implicit assumptions [9,10].

The available research on the digital divide and health issues has focused mainly on access to health-related information [11-15]. Research has also examined how variables such as health status, age, sex, education, and income influence Internet use for health purposes [16-18]. On the other hand, as Dickinson and Gregor [9] showed in their review, the literature that claims that computer and Internet use has a positive effect on the well-being of older people is based on a few studies that do not support that claim. Most of the studies reviewed by Dickinson and Gregor were “intervention” studies with training programs to use computers and the Internet [19,20]. However, the problem with this research is that the effects of computer use, the effects of training, as well as the effects of the context in which computers are used tend to be confounded. Similar problems can be found in more recent studies that claim that Internet training and use contribute to older adults’ well-being [21]. As Dickinson and Gregor noted [9], the improvements reported in these studies may be attributable to the training programs and the social interaction with other learners rather than to computer and Internet use. Other studies reviewed by Dickinson and Gregor, both correlational [22] and qualitative [23], suffered from important limitations (ie, misattribution of causality and inappropriate generalization of results) that question their claims that computer use improves the well-being of older adults. For example, the association reported in some studies between Internet use and health among older people does not indicate the direction of this relationship (ie, people who use the Internet may be healthier, but it is also possible that healthier people are more likely to use the Internet). More recent studies also suffer from selection bias that makes the generalization of results difficult [17,24].

Clearly, more research is needed to explore the relationship between the digital divide and actual indicators of health among older people. The research question we posit is, therefore, whether the digital divide can be considered as a significant determinant of health among older people.The digital divide has often been defined as the split between the “haves” and “have-nots” (or between users and nonusers of new media) [25-27]. This definition has been expanded, however, to include the various dimensions along which inequalities in the digital age may occur [25-28]. Thus, DiMaggio et al [29] refer to the digital divide as the “inequalities in access to the Internet, extent of use, knowledge of search strategies, quality of technical connections and social support, ability to evaluate the quality of information, and diversity of uses” (p. 310). For our purposes, we define the digital divide among older people in terms of Internet users and nonusers.

To our knowledge, this is the first study examining relationships between Internet use and self-rated health among older people using representative samples of Internet users and nonusers from the general population. In this paper we will examine whether Internet use among older people is associated with self-rated health and whether this association holds beyond the socioeconomic position of individuals (ie, the “social divide”), a major social determinant of health [30-37]. It has been suggested that, in addition to age, income and education are two of the most important barriers to Internet use [38-41]. Thus, the inequalities associated with the socioeconomic position of individuals in society are also related to the digital divide [16]. It is possible, therefore, that potential relationships between Internet use and health might be reflecting the relationship between socioeconomic position (a major determinant of access and use of the Internet) and health rather than reflecting benefits of Internet use by itself [3]. This being the case, the relationship between the digital divide and health among older people would be just a reflection of already existing socioeconomic inequalities in health, that is, a reflection of the relationship between the social divide and health.

To disentangle these relationships, we analyzed the association between Internet use and self-rated health, comparing users and nonusers of the Internet between 55 and 74 years of age, taking into account the socioeconomic position of individuals as well as other potential sociodemographic correlates of health: sex, age, marital status, and area of residence.

## Methods

### Study Sample

We used data from a survey about the digital divide and quality of life among older people in Spain conducted in 2008. In Spain, the National Statistics Institute has calculated that, in 2008, there were 1,226,000 Internet users between 55 and 64 years and that this number decreases sharply to 302,000 users between 65 and 74 years [42]. In percentages, 24.4% and 7.9% of people 55-64 and 65-74 years, respectively, had used Internet in the last 3 months. This is 17.3% of the Spanish population between 55 and 74 years. To ensure adequate statistical inference was possible, Internet users were oversampled in the original survey. To do so, the survey takes advantage of two sampling methods to locate eligible participants. Internet nonusers 55-74 years were contacted via random digit dialing and screened about their use of the Internet in the last 3 months. Eligible participants (those not having used the Internet in the last 3 months or more) were interviewed about their health status using computer-assisted telephone interviewing. Response rate for eligible participants (55-74 years) was 60%.

Internet users were sampled from an online research panel of more than 50,000 Spanish Internet users. The recruitment of panel members is based on sociodemographic variables as well as Internet behavior, leading to a high rate of representation of the population of Spanish Internet users. This panel is maintained only for research purposes, with constant recruitment of new members. To exert a tight control of potential sampling bias, eligible participants were selected and invited to participate in the study (targeted advertising), applying quotas of sex, age, size of locality, and education level to match official data [42]. A link to a website containing the online questionnaire and a random identification code were sent to eligible participants by email. The online questionnaire was identical to the telephone interview. This recruiting technique, known as invited participation, allows the researcher to verify that each participant is engaged in the study on one occasion only, and, when combined with targeted advertising, control over sampling is maximized [43,44]. Online participants were given small incentives for completing the questionnaire; no incentive was given to telephone interview participants. Average time to complete the questionnaire was 9 minutes. Once the questionnaire was completed, participants no longer had access to the online survey. Only completed questionnaires were included in the dataset. The response rate, calculated as the ratio between completed questionnaires and emails sent, was 50%. The final sample of Internet users showed only very small deviations from the target population. Small corrections were made in this sample to represent the population of Internet users. For example, 49.6% of those sampled lived in a big city (or surroundings), while the figure in the target population was 49%. For sex, we surveyed 68.4% of men compared to a target of 70%. In all of the remaining categories, the deviations were also very small. According to our data, it seemed that Internet users were self-selected almost completely at random.

The final sample consisted of 709 Spanish individuals between 55 and 74 years and was finally balanced to represent the Spanish population 55-74 years in terms of Internet use and sex across two age groups (55-64 and 65-74 years). Sampling error was ± 3.7% for a 95% confidence interval.

### Outcome Variable

Subjects were asked to rate their health in general on a 5-point scale, ranging from “very good” to “very bad.” We used the categories that fell below “good” health as an indicator for self-rated poor health. This single-item measure of self-rated health is an extensively used measure of health with strong relations with outcomes such as mortality, morbidity, and physical and mental health status across groups with different sociodemographic characteristics, and it has been considered as a valid measure of health [45-48].

### Predictors

Internet use refers to Internet user status (coded as 1 = nonuser, 2 = user) rather than the type of Internet use (ie, frequency). We assigned the status of “user” to those participants who had been connected at least once in the last 3 months. All the remaining participants were considered nonusers. Sex was coded as 1 = male, 2 = female. Age was coded into two groups: 1 = 55-64 years, 2 = 65-74 years. Marital status was coded as 1 = never married, 2 = married/living with partner, 3 = separated/divorced, 4 = widowed. Area of residence was coded as 1 = a country village or farm in the countryside, 2 = a town or small city, and 3 = a big city or the suburbs or outskirts of a big city. These last two were treated as categorical variables in the analyses.

To measure the socioeconomic position of participants, we used an indicator of social class that derives from the cross-classification of occupation and educational attainment of the head of family (main income earner). This cross-classification is a standard for media studies in Spain and provides five different social classes (high, medium-high, medium, medium-low, and low) by combining head of family education level and occupation (or last occupation) [49]. Given that education level and occupation were used for the computation of social class, this information was not used separately in the statistical analysis, to avoid multicollinearity.

### Analytical Strategy

For the analysis of the data, we used multivariate binomial logistic regression to estimate the odds ratios of being in the self-rated poor health category. We estimated four regression equations (models) in a nested fashion. The first equation (Model 1) tested whether there was any association between Internet use and health. Model 2 adds sociodemographic covariates (sex, age, and marital status) to equation 1. In Model 3, we included area of residence. Finally, in Model 4, we included social class as a covariate to estimate the effect of Internet use on health, controlling for socioeconomic effects. Odds ratios, 95% confidence intervals, deviation statistics, and chi-square values were calculated for each model.

## Results

Table 1 presents descriptive statistics of the study participants.

**Table 1:** Descriptive statistics of study participants

Variable	No.	%
**Sex**		
Female	370	52.2
**Age**		
65-74 years	307	43.3
**Marital status**		
Never married	22	3.2
Married/living with partner	564	79.6
Separated/divorced	47	6.6
Widowed	76	10.6
**Area of residence**		
Country village or farm	296	41.8
Town or small city	145	20.4
Big city or surroundings	268	37.8
**Social class**		
High	57	8.1
Medium-high	65	9.2
Medium	186	26.3
Medium-low	251	35.5
Low	149	21.0
**Internet use**		
User	123	17.3
**Self-rated health**		
Poor	119	16.8

Table 2 summarizes the covariates of self-rated poor health from the four binomial logistic regressions models.

Results for Model 1 show that Internet users have statistically significant lower odds of being in the poor health category as compared to nonusers. This result remained for Model 2 and Model 3 as well, indicating that the effect of Internet use on health was still present after taking into account sex, age, marital status (Model 2), and area of residence (Model 3). In the specific case of marital status, we further checked if the small size of the “never married” category was affecting the results. Results remained the same whether we collapsed marital status into married vs other, or any other combination.

The inclusion of social class as a continuous covariate in Model 4, however, removed the statistical significance of the influence of Internet use on health that was observed in previous models (OR = 0.61, *P* = .23).

The only remaining significant covariate in Model 4 other than socioeconomic position was sex, indicating that women have 1.90 greater odds of being in the poor health category than men (*P* = .004), after adjusting for all other covariates of the study.

**Table 2:** Covariates of self-rated poor health from four binomial logistic regressions models

	Model 1	Model 2	Model 3	Model 4
	OR (95% CI), *P*	OR (95% CI), *P*	OR (95% CI), *P*	OR (95% CI), *P*
**Internet user status**				
Nonuser	1	1	1	1
User	0.32 (0.16-0.67), .002	0.39 (0.18-0.83), .01	0.41 (0.19-0.87), .02	0.61 (0.27-1.37), .23
**Sex**				
Male		1	1	1
Female		1.87 (1.22-2.89), .004	1.89 (1.23-2.91), .004	1.90 (1.23-2.92), .004
**Age**				
55-64 years		1	1	1
> 64 years		0.99 (0.65-1.45), .99	0.97 (0.64-1.47), .88	0.95 (0.63-1.45), .82
**Marital status**				
Never married		1	1	1
Married/living with partner		1.53 (0.41-5.74), .52	1.45 (0.39-5.46), .58	1.43 (0.38-5.42), .60
Separated/divorced		0.64 (0.23-3.32), .59	0.65 (0.12-3.38), .61	0.62 (0.26-3.27), .57
Widowed		0.69 (0.16-3.03), .62	0.66 (0.15-2.91), .58	0.61 (0.14-2.73), .52
**Area of residence**				
Country village or farm			1	1
Town or small city			0.72 (0.40-1.32), .29	0.82 (0.45-1.51), .53
Big city or surroundings			1.02 (0.66-1.60), .90	1.14 (0.73-1.80), .57
**Social class**				0.74 (0.59-0.92), .008
				
Constant	0.23, < .001	0.12, .002	0.13, .004	0.24, .05
χ^2^ (df)	12.17 (1), < .001	25.84 (6), < .001	27.31 (8), < .001	34.82 (9), < .001
Model deviation^a^	630.02	616.35	614.89	607.37

^a^ Model deviation is measured as −2 log likelihood.

## Discussion

This paper presents analyses from cross-sectional data exploring the potential association between Internet use and self-rated health among older people. Results initially showed a significant relationship between Internet use and self-rated health (Model 1), suggesting that Internet users have better self-rated health than nonusers. This effect remained when other sociodemographic variables (sex, age, marital status, and area of residence) were entered into the equation (Models 2 and 3). However, the significant relationship between Internet use and self-rated health disappeared once social class was considered (Model 4). Overall, these results suggest that there is no evidence supporting the idea that use of the Internet has a significant relationship with health for the older population once the socioeconomic position of individuals is taken into account.

The analysis of Internet users aged 55-74 years in relation to health issues is a strength of the study. Traditionally, little attention has been paid to Internet users in this age group. For instance, in Spain, little is known about this segment of the population beyond the fact that they constitute a rather small group. It has been suggested that access to and participation in the information society among older people will promote positive outcomes in health and well-being [3,6-9]. From this viewpoint, the digital divide would be a significant determinant of health for older people. And it appears to be so when the social position of individuals is ignored. Our results suggest, however, that the digital divide is not a source of health inequalities beyond already-existing socioeconomic inequalities of health. Therefore, the apparent relationship between the digital divide and health among older people appears to be a reflection of existing social inequalities in health. In other terms, Internet users can be healthier provided that they are wealthier. In this regard, our study further illustrates the association between socioeconomic position and heath indicators [30-37]. The socioeconomic gradient in health is a well-established finding in the literature that, even though it declines with age [45,46], extends to older people [34,47,50]. Furthermore, this socioeconomic gradient in health is observed regardless of whether socioeconomic status is measured by occupation, education, or income [35,37,47]. Our results also revealed gender differences in self-rated health that are in line with other studies reporting higher proportions of women rating their health as poor [33,50-54].

### Limitations

The study has several limitations. First, we examined self-rated health (ie, perceptions of health in general) and did not include specific measures of mental health. Future research would benefit from including specific measures of physical and mental health. Second, recent research has shown how self-rated health responses, our outcome variable, might be biased in certain sociodemographic groups. For instance, Delpierre et al [32] have shown that the impact of health problems on self-rated health is stronger among better-educated individuals. This phenomenon could lead to an underestimate of the health inequalities across socioeconomic groups. In our study, social class behaved as a key determinant of health among Internet users and nonusers, and, according to Delpierre et al, we cannot be sure about the real difference in health. Future research focusing on other measures of health is clearly needed. Third, random sampling of Internet users was done according to official data about people 55-74 years who used the Internet in the last 3 months. This is a broad definition of an Internet user that might have an effect on the results of the study. Finally, some caution must be taken in generalizing our results. Our data refer to cohorts of older people (individuals born between 1934 and 1953) with relatively small exposure to the Internet and other tools of the information society. It remains to be seen whether, for future cohorts of older people with greater exposure to the information society, the digital divide becomes a significant source of health inequalities. This is certainly an issue that deserves further research and consideration. In this context, future studies should also examine whether, among Internet users, those in higher socioeconomic groups would achieve better health outcomes through better information use and better use of the Internet.

### Conclusions

In conclusion, results from this paper suggest that beyond the social divide, the digital divide does not add another source of health inequalities for older people. Older people are among the groups most excluded from the information society. Reducing the digital divide among older people has become a target for many policy initiatives since it is believed that the information society will provide benefits for the well-being of older people [9,10]. However, as the digital divide is also an expression of social inequalities, policies and initiatives aiming to reduce the digital divide, without reducing the social divide, may contribute to existing socioeconomic inequalities and may benefit those already advantaged.
